# Editorial: Psychological sleep studies: new insights to support and integrate clinical practice within the healthcare system, volume II

**DOI:** 10.3389/fpsyg.2024.1525122

**Published:** 2024-12-03

**Authors:** Marco Sforza, Luigi Ferini-Strambi, Christian Franceschini

**Affiliations:** ^1^Department of Clinical Neurosciences, San Raffaele Scientific Institute (IRCCS), Milan, Lombardy, Italy; ^2^Department of Clinical Neurosciences, Neurology-Sleep Disorders Center, IRCCS San Raffaele Scientific Institute, Milan, Italy; ^3^Department of Medicine and Surgery, University of Parma, Parma, Emilia-Romagna, Italy

**Keywords:** sleep health, insomnia management, mental health and sleep, cognitive-behavioral therapy for insomnia (CBT-I), telehealth interventions, sleep disorders and psychological wellbeing, sleep quality of healthcare professionals, clinical psychology

## Introduction

Sleep health is essential for overall physical and mental wellbeing. Insufficient sleep, whether in terms of quantity or quality, significantly impacts an individual's quality of life and day-to-day functioning (Ramar et al., 2021). Consequences of sleep disorders like insomnia include impaired daytime cognition, reduced work productivity, an increased risk of accidents and injuries, and have been linked to psychiatric disorders, cardiovascular diseases, and other chronic health conditions (Baglioni et al., 2011; Kyle et al., 2010a,b; Rössler et al., 2018; Vgontzas et al., 2010; Fernandez-Mendoza et al., 2010; Vgontzas et al., 2009; Laugsand et al., 2011; Daley et al., 2009; Sivertsen et al., 2006; Hafner et al., 2023; Léger et al., 2014; Erickson et al., 2017; Garbarino et al., 2017; Hertenstein et al., 2019).

Sleep problems may compromise with the treatments that are currently being administered for a wide range of illnesses. When these disorders are addressed, they have the potential to enhance the patient's quality of life and adherence to therapy, as well as the intensity of the psychological symptoms that they are experiencing. It has been shown that there is a complicated association between sleep and mental health issues, such as anxiety, depression, and traumatic stress disorders. This interaction is considered to be bidirectional (Baglioni et al., 2011; Hertenstein et al., 2019; Sivertsen et al., 2012). There is a growing demand in the clinical setting for psychologists who are skilled in evidence-based psychotherapy to become members of multidisciplinary health teams. These psychologists would be tasked with providing cognitive-behavioral intervention and support for patients suffering from insomnia (Baglioni et al., 2020), narcolepsy (Agudelo et al., 2014; Franceschini et al., 2021), and adherence to treatment for obstructive sleep apnea (Sforza et al., 2024).

This volume brings together a collection of research that delves into different aspects of sleep health and its relationship with psychological wellbeing, offering new approaches to addressing these challenges in various populations. The findings provide critical implications for clinical practice, aiming to improve sleep health management across diverse healthcare settings.

## Mental health and sleep quality in healthcare practitioners during COVID-19

Luo et al. investigate the mental health challenges, particularly anxiety and poor sleep, experienced by healthcare workers during the COVID-19 pandemic. Their study shows that Progressive Muscle Relaxation (PMR) significantly alleviates anxiety and improves sleep quality among practitioners in high-stress environments like mobile cabin hospitals. The intervention demonstrated a reduction in both anxiety and poor sleep indices, offering an accessible and effective solution to mitigate mental health strains during pandemic-related crises.

### Implications

This study highlights the potential of PMR as a non-invasive, cost-effective intervention that could be readily implemented in healthcare systems to support the mental health of frontline workers. Given the global scale of the healthcare crisis, integrating such tools into standard healthcare practice could enhance workforce wellbeing and performance.

## Insomnia management: patient journey across Europe and Canada

In their study, O'Regan et al. explore the patient journey in managing insomnia across Europe and Canada. By mapping the phases of insomnia management, from self-initiated behavioral changes to long-term reliance on prescription medications, the authors reveal a considerable gap in the alignment of healthcare provider strategies and patient needs. Their research uncovers key points where interventions can be improved, particularly in addressing patient expectations and enhancing education around sleep hygiene and non-pharmacological treatments.

### Implications

Overall, the findings show that patient education is crucial to remove the stigma surrounding insomnia, helping patients recognize that chronic insomnia is a primary disorder that requires appropriate medical management, and encouraging them to seek help earlier. Additionally, healthcare provider education and training on sleep disorders are essential to increase the perception of chronic insomnia as a serious primary medical condition and to fully understand its significant impact on patients' daily lives.

Furthermore, the current situation, in which many patients must settle for “manageable” insomnia, highlights a serious unmet need regarding the management and treatment of this condition. More comprehensive approaches are needed to ensure that patients receive effective care that goes beyond symptom management, addressing the root causes of their insomnia and improving their long-term health outcomes.

## Prevalence and predictors of insomnia in university students

Wale et al. shed light on the high prevalence of insomnia among university students in Ethiopia. Their study identifies key predictors, including gender, age, mild anxiety symptoms, and excessive use of mobile devices before bedtime, which significantly contribute to poor sleep quality among students. The findings underscore the impact of lifestyle factors and mental health challenges on sleep patterns in young adults, a group particularly vulnerable to sleep disturbances due to academic stress and lifestyle transitions.

### Implications

The study calls for targeted interventions focusing on mental health support and promoting better sleep hygiene among university students. Educational institutions should integrate sleep health education into their wellness programs, emphasizing the importance of mental health and limiting late-night screen exposure.

## Telehealth delivery of cognitive-behavioral therapy for insomnia for chronic pain patients

Zambelli et al. evaluate the feasibility of delivering CBT-I through telehealth to chronic pain patients suffering from insomnia. The study shows that adapted CBT-I delivered via telehealth not only improved sleep quality but also alleviated symptoms of anxiety, and depression. The research highlights the growing role of telehealth in making effective sleep interventions more accessible, particularly for populations with mobility issues or those living in remote areas.

### Implications

Telehealth-based interventions offer a scalable and practical solution to reach populations with limited access to in-person therapy. As healthcare systems continue to embrace digital health solutions, telehealth-based CBT-I can play a pivotal role in addressing sleep disorders, particularly in the post-pandemic era where telehealth has become more prominent.

## Future directions in sleep research

While the studies presented in this volume offer important insights, several areas remain unexplored, signaling opportunities for future research. First, the long-term effectiveness of non-pharmacological interventions like PMR and CBT-I across diverse populations, including adolescents and the elderly, needs further investigation. Additionally, the impact of behavioral and lifestyle factors, such as mobile device use before bedtime, which has been shown to significantly influence sleep quality, particularly among younger populations, requires deeper examination.

As telehealth becomes an increasingly central component of healthcare delivery, there is also a need to assess its broader efficacy across varying cultural contexts, socioeconomic groups, and medical conditions. Understanding how digital interventions like telehealth-delivered CBT-I can be optimized to meet the needs of different populations will be crucial for the equitable expansion of these services.

## Conclusion: toward a holistic approach to sleep and mental health

The collection of research in this volume underscores the profound interconnectedness of sleep health and psychological wellbeing. From frontline healthcare workers grappling with anxiety during pandemics to chronic pain patients benefiting from telehealth-delivered CBT-I, these studies highlight the necessity of integrating sleep health into broader mental healthcare strategies. Sleep disorders, particularly insomnia, are often underdiagnosed and undertreated, despite their substantial repercussions on both individual and societal health.

Psychologists play a crucial role in addressing sleep-related disorders, not only through therapeutic interventions such as CBT-I but also by promoting sleep hygiene and mental wellness as foundational elements of health. Academic training programs in psychology should emphasize the importance of sleep as a pillar of mental wellbeing, equipping future practitioners with the skills to recognize, address, and treat sleep disturbances as part of a comprehensive mental health approach.

To advance a holistic approach, healthcare systems must prioritize early detection and treatment of sleep disorders, leveraging cross-disciplinary methods that integrate mental health, technology, and lifestyle interventions. This volume provides a robust foundation for understanding the significance of sleep in clinical practice, yet ongoing research and innovation will be essential to refine and expand these approaches, fostering a truly integrative model of mental healthcare that includes sleep as a central component.
